# Discriminating Epithelial to Mesenchymal Transition Phenotypes in Circulating Tumor Cells Isolated from Advanced Gastrointestinal Cancer Patients

**DOI:** 10.3390/cells11030376

**Published:** 2022-01-22

**Authors:** Adriana Carneiro, Paulina Piairo, Alexandra Teixeira, Dylan Ferreira, Sofia Cotton, Carolina Rodrigues, Alexandre Chícharo, Sara Abalde-Cela, Lúcio Lara Santos, Luís Lima, Lorena Diéguez

**Affiliations:** 1 International Iberian Nanotechnology Laboratory, Avenida Mestre José Veiga s/n, 4715-330 Braga, Portugal; adriana.carneiro@inl.int (A.C.); alexandra.teixeira@inl.int (A.T.); carolina.rodrigues@inl.int (C.R.); alexandre.chicharo@inl.int (A.C.); sara.abalde@inl.int (S.A.-C.); 2 IPO Experimental Pathology and Therapeutics Group, Research Center of IPO Porto (CI-IPOP)/RISE@CI-IPOP (Health Research Network), Portuguese Oncology Institute of Porto (IPO Porto), Porto Comprehensive Cancer Center (Porto.CCC), 4200-072 Porto, Portugal; i38795@ipoporto.min-saude.pt (D.F.); sofia.ribeiro.cotton@ipoporto.min-saude.pt (S.C.); lucio.santos@ipoporto.min-saude.pt (L.L.S.); luis.carlos.lima@ipoporto.min-saude.pt (L.L.); 3 Department of Surgical Oncology, Portuguese Institute of Oncology (IPO Porto), 4200-072 Porto, Portugal

**Keywords:** circulating tumor cells, gastrointestinal cancer, epithelial-to-mesenchymal transition, metastasis, liquid biopsy, microfluidics

## Abstract

Gastrointestinal (GI) cancers constitute a group of highest morbidity worldwide, with colorectal cancer (CRC) and gastric cancer being among the most frequently diagnosed. The majority of gastrointestinal cancer patients already present metastasis by the time of diagnosis, which is widely associated with cancer-related death. Accumulating evidence suggests that epithelial-to-mesenchymal transition (EMT) in cancer promotes circulating tumor cell (CTCs) formation, which ultimately drives metastasis development. These cells have emerged as a fundamental tool for cancer diagnosis and monitoring, as they reflect tumor heterogeneity and the clonal evolution of cancer in real-time. In particular, EMT phenotypes are commonly associated with therapy resistance. Thus, capturing these CTCs is expected to reveal important clinical information. However, currently available CTC isolation approaches are suboptimal and are often targeted to capture epithelial CTCs, leading to the loss of EMT or mesenchymal CTCs. Here, we describe size-based CTCs isolation using the RUBYchip™, a label-free microfluidic device, aiming to detect EMT biomarkers in CTCs from whole blood samples of GI cancer patients. We found that, for most cases, the mesenchymal phenotype was predominant, and in fact a considerable fraction of isolated CTCs did not express epithelial markers. The RUBYchip™ can overcome the limitations of label-dependent technologies and improve the identification of CTC subpopulations that may be related to different clinical outcomes.

## 1. Introduction

Gastrointestinal (GI) cancers can develop from any anatomic sites of the digestive system, ranking as some of the most frequently diagnosed cancer types worldwide and constituting a group of highest morbidity [1]. Overall, in gastrointestinal cancers, the patient prognosis differs significantly depending on the disease stage at the time of diagnosis. Due to screening campaigns during recent years, survival rates have been extended by earlier diagnosis and improved surgical techniques. Still, a considerable number of cases are diagnosed late, mostly due to a general lack of symptoms in the early stages of the disease, and in those cases, if the disease has progressed to an unresectable stage, the prognosis remains unfavorable [1,2,3].

Indeed, out of all cancer types, colorectal is the third most commonly diagnosed and the second with the highest mortality worldwide. Among GI cancer types, liver cancer is the second with the highest mortality, followed by gastric, and esophageal cancer (EC). Importantly, a considerable portion of early-stage GI cancer patients who undergo curative resection suffer metastatic disease within 5 years of surgery [4]. In gastric cancer in particular, approximately half of the patients develop metastasis within the first 5 years after surgery or suffer from severe treatment side-effects [5]. Hence, this might indicate that either an occult metastatic process is occurring in parallel with primary tumor development (that went undetected through conventional diagnostic methodologies), or that tumor cells with metastatic potential have entered the bloodstream, ultimately causing subsequent local and/or distant metastasis [6,7].

These cells, termed circulating tumor cells (CTCs), are defined as extremely rare malignant cells that are shed from the primary or metastatic tumors into the bloodstream. Even though CTCs have a short half-life in circulation, they can extravasate and form metastasis [6]. As such, their identification, enumeration and molecular characterization can provide valuable information. CTCs have been proposed to be used as an easy screening tool to facilitate early diagnosis of malignant diseases [8]. Moreover, CTC detection in solid tumors can improve the accuracy of prognosis, extend the risk stratification for patient with occult micrometastases and help to provide the best individual therapy to every patient [8]. Downstream analysis of CTCs from each cancer patient will allow the identification of therapeutic targets to guide patient treatment and to monitor molecular evolution of the disease, ultimately allowing the study of cell clonality and the discovery of new biomarkers [8,9]. Actually, a recent study in CRC has already confirmed that CTC line profiling is a relevant approach to study clonal selection during disease progression, as well as to discover new CTC biomarkers for monitoring treatment response [10].

A number of studies have been conducted in order to evaluate the relevance of CTC enumeration in GI related malignancies. Regarding gastric cancer, CTC enumeration and their molecular profiling have been suggested to be useful biomarkers for diagnosis [11]. In addition, most studies demonstrated a clear association between higher CTC counts and worse prognosis in colorectal, pancreatic (PC) and liver cancer [12,13,14]. Furthermore, preliminary studies on CTC status before and after surgery in esophageal cancer patients also demonstrated that the presence of these cells was an independent predictor of disease recurrence [13].

Besides CTCs enumeration, distinguishing CTCs subtypes by assessing their phenotype could contribute to a better understanding of crucial aspects of metastization and could foster personalized medicine approaches [15]. Epithelial-to-mesenchymal transition (EMT) has long been known as a crucial mechanism in the metastatic process, generating various hybrid phenotypes along the epithelial to mesenchymal (M) differentiation axis, and thereby increasing tumor heterogeneity. It is also reported that cooperative processes between different phenotypes may also occur, by which EMT-shifted cells would help more epithelial phenotypes to increase and survive in the circulation, and possibly find niches in secondary organs [16,17]. Several studies have already revealed EMT-associated heterogeneity in the CTC population, showing the presence of mesenchymal and epithelial CTCs and correlating the different phenotypes with different clinical outcomes, stages, and metastases, not only in GI cancers [18,19,20,21], but in other cancer types as well [22,23].

**Figure 1:**
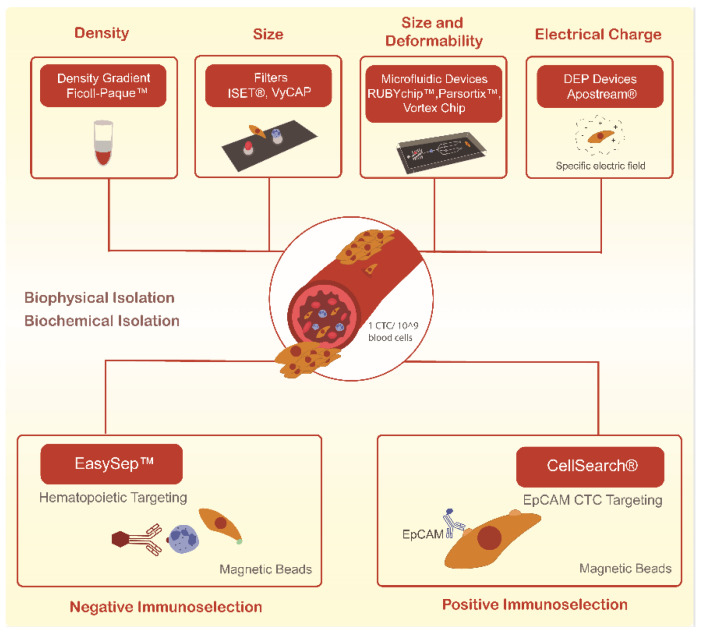
Current methodologies used to isolate circulating tumor cells (CTCs) through biological and biophysical properties.

Still, CTCs capture remains technically challenging due to their low abundance in a background of millions of blood cells, even in metastatic settings, and also due to their many differences across tumor types [24]. As such, the clinical utility of CTCs has been hampered by the difficulty of consistently isolating the different subpopulations of these cells. More recently, many techniques have been developed for the reliable isolation of CTCs. These mainly rely on either of two approaches—biochemical and biophysical isolation, as schematized in Figure 1. Biochemical isolation is based on the identification of unique biomarkers, while biophysical isolation approaches rely on the differentiation between the intrinsic physical properties of CTCs and blood cells [25].

To date, the only CTCs isolation method cleared by the Food and Drug Administration (FDA) is the CellSearch^®^ system. This biochemical isolation system operates through immunomagnetic-conjugated antibodies against EpCAM, a transmembrane protein present in some CTCs, but absent in blood cells. However, some CTCs, especially those of a highly invasive and metastatic capacity, do not express these antigens, which suggests that significant cell loss during the CTCs capture step may be occurring [26]. In addition, this technology relies on a multi-step protocol, is labor-intensive, and only provides CTCs detection and enumeration [26,27].

As an alternative, and based on the fact that CTCs are physically distinct from most normal blood cells, several technologies to isolate CTCs based on their physical properties have gained interest. Indeed, on average CTCs are larger than white blood cells (8–20 µm), allowing the development of size-based approaches, including membrane filters and a wide variety of microfluidic devices [28]. This type of systems allow a straightforward process of sample loading, separation, and the capture of living rare cells in one single step that can be analyzed in situ or downstream through cellular, microscopic or molecular techniques, or even recovered for functional studies [29,30].

Our research group has previously developed and validated microfluidic systems for the efficient and rapid isolation of unfixed CTCs, the later being the RUBYchip™. These systems have been demonstrated to capture CTCs based on their size and deformability directly from a whole blood sample in different cancer types, including colorectal, breast and bladder cancer [31,32,33,34]. In this work, using this microfluidic device, we were able to efficiently isolate CTCs in all samples from 11 advanced GI cancer patients and to classify them according to their expression of cytokeratin (CK) and Vimentin (VIM). As such, we demonstrated the potential of this technology to capture CTCs of different phenotypes, even those undergoing epithelial–to-mesenchymal transition (EMT), which are potentially relevant in metastatic dissemination.

## 2. Materials and Methods

### 2.1. RUBYchip™ Microfluidic Device Design and Fabrication

Briefly, RUBYchip™ is a microfluidic device able to process 7.5 mL of whole blood. As previously described [34], it first separates the sample into two principal areas, each containing four separated modules. Each module features a single row of anisotropic micropillars interspaced 5 μm, comprising the cell filtering area. The size, geometry, and aspect ratio of the micropillars were carefully chosen to allow blood cells to deform and gently flow through, while larger and more rigid cells are retained. The microfluidic masters were designed in AutoCAD software and fabricated on a silicon wafer using photolithography and deep reactive ion etching, as described elsewhere [31]. To assemble the microfluidic device, conventional soft lithography processes were employed, using polydimethylsiloxane (PDMS). In short, the prepolymer was mixed with the cross-linker (SYLGARD^TM^ 184 Silicone Elastomer, Ellsworth Adhesives Iberica, Madrid, Spain) and poured onto the master mold, degassed, and cured. Once peeled from the master mold, the PDMS replica was irreversibly bonded to a standard glass slide (25 × 75 mm^2^, ThermoFisher Scientific, Darmstard, Germany) using oxygen plasma (Plasma Cleaner PDC-002-CE, Harrick Plasma, Ithaca, NY, USA). To operate the device, flow was driven from the inlet to the outlet through a syringe pump (NE-1200, New Era Syringe Pumps, Farmingdale, NY, USA). Then, the devices were primed using 250 µL of Ethanol (Sigma Aldrich/Merck, KGaA, Darmstadt, Germany), 250 µL of 10 mM Phosphate Buffer Saline (PBS, Sigma Aldrich), and 250 µL of 1% Pluronic F-127 (Sigma Aldrich).

### 2.2. Cell Culture

Several human GI cancer cell lines were cultured, including human colorectal (SW480, SW620, Caco-2, HT-29), gastric (AGS, N87, OCUM-1), esophageal (Kyse 30) and pancreatic (PANC-1, BxPC-3). The N87, AGS and Kyse 30 cells were grown in RPMI-1640 medium, whereas the CRC and pancreatic cell lines were cultured in Dulbecco’s Modified Eagle’s Medium (DMEM, Gibco, ThermoFisher Scientific, Darmstard, Germany). All cell lines were regularly tested for mycoplasma every 6 months (Venor^®^GeM Classic, Minerva Biolabs, Berlin, Germany). Both media were supplemented with 1% Penicillin/Streptomycin (Pen/Strep, Corning, Inc., Corning, NY, USA) and 10% Fetal Bovine Serum (FBS, Gibco) except for Caco-2, which required a 20% FBS supplementation. The OCUM-1 cells were grown in low glucose DMEM supplemented with 1% non-essential amino acids (NEAA). All cell lines were cultured at 37 °C in a 5% CO_2_ atmosphere.

### 2.3. Spiking Experiments and Whole Blood Processing

Adherent cells were harvested by incubation with 0.25% Trypsin-EDTA to obtain a cell suspension, then resuspended in complete growth media, incubated with Hoechst for 30 min to stain the nucleus and finally spiked (200 cells) in 7. 5 mL of whole blood samples from healthy donors, prior to injection in the device. Simultaneously, the same number of cells (200 cells) were added to a well-plate as the control of the spiked cells. To determine the optimal capture efficiency of the microfluidic device, six different flow rates were tested, including 60, 80, 100, 120, 160 and 200 µL/min. All experiments were performed using triplicates. Trapped cells were rinsed with 2% Bovine Serum Albumin (BSA, Sigma Aldrich), fixed with 4% Paraformaldehyde (PFA, Sigma Aldrich) for 20 min at room temperature (RT) and washed with PBS. Both the device and the well-plate were scanned using a fluorescence microscope (Nikon Eclipse Ti-E microscope, Nikon, Amsterdam, Netherlands) and Hoechst-positive cells were counted to determine the number of trapped cells inside the device and the total number of spiked cells. Capture efficiency was calculated as the number of trapped cells over the total number of target cells spiked into the initial whole blood sample, as already described [34].

### 2.4. Cell Size and Nucleus-to-Cytoplasm Ratio

**Figure 2:**
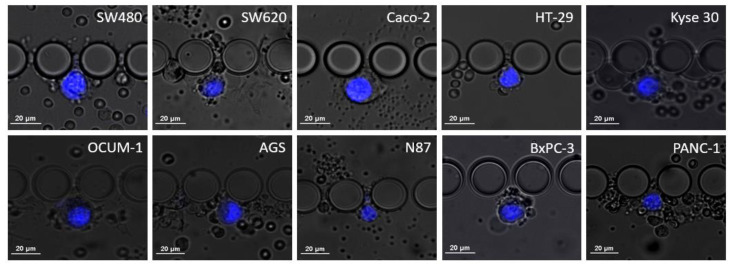
Microscopic images of the different cell lines when trapped inside the RUBYchip™ obtained after spiking experiments and stained with DAPI. The images were acquired using a 20× objective and used to calculate average cell dimensions and study cell morphology.

Cell dimensions, particularly cell and nucleus diameters, were assessed using NIS Elements analysis software (Nikon, Amsterdam, The Netherlands), measurements were estimated from images obtained from spiking experiments, as shown in Figure 2. Then, the nucleus-to-cytoplasm (NC) ratio was calculated as the cell nucleus diameter over the cell diameter.

### 2.5. Immunocytochemistry Studies

CTC identification was achieved by immunofluorescence, and several experimental conditions were tested to optimize antibody staining conditions. Adherent cells (45,000 to 150,000 cells, depending on cell line) were firstly seeded onto sterile cover slips, previously treated with Poly-l-Lysine (Sigma Aldrich) and left to grow for 48 or 72 h. In this study, peripheral blood mononucleated cells (PBMCS) isolated by density gradient centrifugation (Histopaque^®^, Sigma Aldrich) from the blood of healthy donors were used as a negative control. The selected monoclonal antibodies were anti-pan Cytokeratin FITC antibody (clone C-11, recognizing human cytokeratins 4, 5, 6, 8, 10, 13 and 18, Sigma Aldrich), monoclonal anti-Vimentin eFluor 570 (Clone V9, Thermo Fisher Scientific), and, lastly, a monoclonal Anti-CD45 Alexa Fluor 647 (Santa Cruz Biotechnology, Heidelberg, Germany), allowing the identification of white blood cells (WBC). Additionally, DAPI (NucBlue™; Invitrogen, MA, USA) was used to stain the cell nucleus. The adherent cells were first permeabilized with 0.25% Triton X-100 solution (Sigma Aldrich), washed with PBS and blocked with 2% BSA. Subsequently, cells were fluorescently labeled with the selected cocktail of antibodies for 1 h at RT. After the incubation period, cells were washed first with 0.5% BSA and then PBS. The same immunocytochemistry protocol was applied to the PBMCs. A similar ICC protocol was performed on the RUBYchip™ using spiked blood to mimic the conditions of clinical samples. For that, approximately 200 cells of the selected cell lines were spiked in 7.5 mL of whole blood from healthy volunteers. In order to fix, permeabilize, block, stain and wash the cells trapped inside the device, all reagent solutions were pumped at the predetermined flow rate using a syringe pump. After cell isolation and staining, multi-channel fluorescence images were acquired using a fluorescence microscope. The presence of DAPI, CK, VIM and CD45 were analyzed in the blue, green, orange, and red channels, respectively.

### 2.6. Gastrointestinal Cancer Patient Samples

In this proof-of-concept study, 11 advanced GI cancer patients (3 CRC, 3 GC, 3 EC and 2 PC) were recruited at Instituto Português de Oncologia do Porto (IPO-Porto), after providing written informed consent. This study was approved by the responsible Ethics Committee at IPO-Porto and following international guidelines. Whole blood samples (7.5 mL) were collected in EDTA-coated tubes and then shipped to the International Iberian Nanotechnology Laboratory (INL) to be processed in the RUBYchip™ within four to six hours.

### 2.7. CTC Isolation and Characterization in Clinical Samples

**Figure 3:**
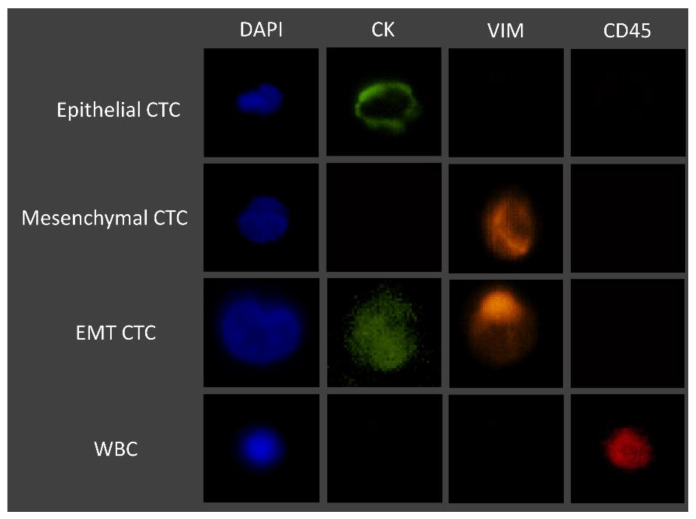
Immunofluorescence staining characteristics for the identification of CTCs.

Whole blood patient samples were processed using the RUBYchip™. Trapped cells were fixed with 4% PFA for 20 minutes, permeabilized with 0.25% *v*/*v* Triton X-100 for 10 min, blocked with 2% BSA for 30 min, and labelled for 1 h at RT with the selected cocktail of antibodies. Once staining was finalized, patient samples were imaged using a fluorescence microscope, and CTCs were identified and manually enumerated using specific classification criteria, as summarized in Figure 3. Gastric, esophageal, and pancreatic cancer samples were imaged and analyzed using an automated system (Allegro Plus, Bioview, Rehovot, Israel) for image acquisition and software-guided CTC enumeration and phenotyping, using the same criteria. Briefly, CTCs were defined as nucleated, determined by DAPI staining, and expressing either CK and/or VIM and lacking the marker of hematopoietic lineage CD45 (CK+/CD45− or VIM+/CD45−).

## 3. Results

### 3.1. RUBYchip™ Performance Assessment Using Human Gastrointestinal Cell Lines

In order to assess the efficiency of the RUBYchip™ for the isolation of gastrointestinal cancer cells, several human GI cancer cell lines were used. Since RUBYchip™ CTC isolation relies on cell size and deformability, we began by performing the morphological characterization of the selected cell lines. Cell dimensions such as cell size and NC ratio were assessed on cells in suspension trapped inside the microfluidic device. Within the ten tested cell lines, as expected, cell size variation was observed. The averaged cell size and NC ratio are presented in Table 1.

**Figure 4:**
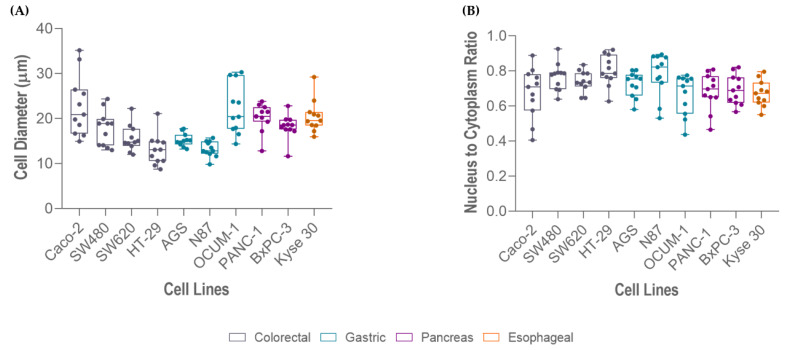
Measurements of different gastrointestinal cancer cell lines (Caco-2, OCUM 1, Kyse 30, PANC-1, BxPC-3, SW480, SW620, AGS, N87, HT-29): (**A**) cell size, and (**B**) nucleus-to-cytoplasm (NC) ratio.

The average cell size of the selected cell lines ranged from 13 to 23 µm in diameter, approximately. Measurements showed that HT-29 were the smallest cells (13.03 µm) and Caco-2 were the largest (22.80 µm). In agreement with size, HT-29 cells presented the highest NC ratio (0.80), whereas Caco-2 (0.68), OCUM 1 (0.67) and Kyse 30 (0.67) presented the lowest, as represented in Figure 4.

**Figure 5:**
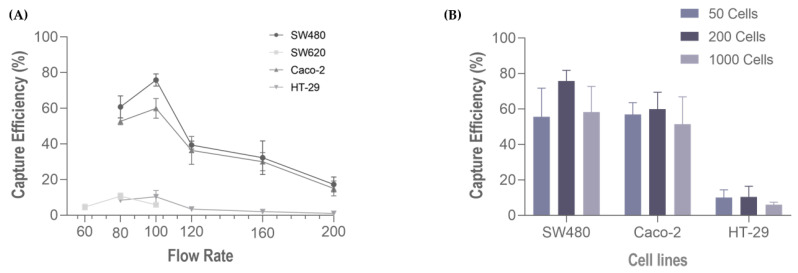
Capture efficiency of the RUBYchip™ (**A**) at six flow rates, using four different CRC cell lines (SW680, Caco 2, SW620 and HT-29), and (**B**) varying the number of spiked cells (50, 200 or 1000) at 100 µL/min, with three different cell lines, SW480, Caco 2.

Following cell characterization, spiking experiments were performed using several cell lines comprising all cancer models (colorectal, gastric, esophageal and pancreatic), these represent the different phenotypic profiles within the epithelial-mesenchymal transition (EMT) spectrum. We first evaluated the performance of the RUBYchip™ to isolate cells of CRC origin, using four different cell lines (Caco-2, SW480, SW620 and HT-29). Six flow rates were tested to optimize the sample processing: 60, 80, 100, 120, 160 and 200 µL/min. The results from the spiking experiments consistently demonstrated that CRC cell lines had higher capture efficiency when applying a flow rate of 100 µL/min, except SW620 cells which showed the highest capture efficiency at 80 µL/min. At the optimal flow rate (100 µL/min), the SW480 cells achieved the highest capture efficiency, 70.20% ± 10.70 (average, N = 4), followed by Caco-2 (60.0% ± 5.9; N = 3), HT-29 (10.30% ± 5.0; N = 3) and, lastly SW620 (5.90% ± 1.3; N = 3) (Figure 5A). To evaluate the influence of the number of spiked cells in the capture efficiency of the microfluidic device, different quantities of CRC cells (50, 200, 1000 cells) were spiked into 7.5 mL of whole blood samples from healthy volunteers and processed in the device at the optimal flow rate, 100 µL/min, previously determined. These experiments revealed that there was no significative difference in the capture efficiency when changing the spiked cell target, since the capture efficiencies remained unaltered: high (60–70%) in SW480 and Caco-2 and low (6–10%) in HT-29 (Figure 5B).

**Figure 6:**
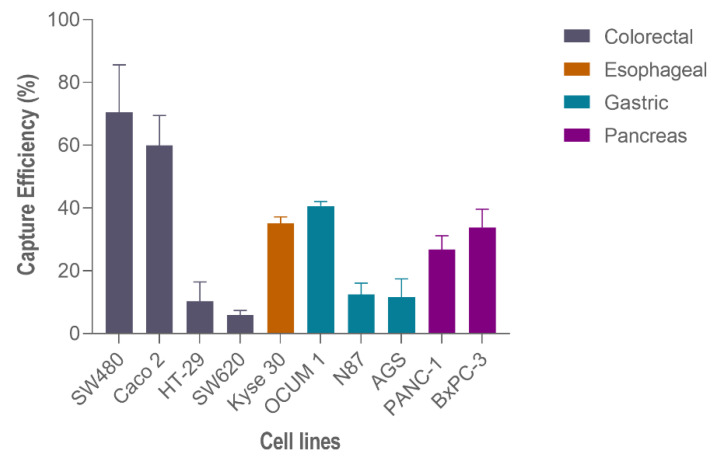
Capture efficiency of the RUBYchip™ using different gastrointestinal cancer cell lines.

Hence, to perform capture efficiency assessment in other GI cancer models, spiking experiments in additional cell lines were carried out using 200 spiked cells and 100 μL/min (Figure 6). In particular, regarding gastric cancer cell lines, the highest capture efficiency was observed on OCUM-1 cells (40.7% ± 1.2, N = 3), followed by N87 cells (12.50 % ± 2.9, N = 3) and finally AGS cells (11.6% ± 4.8, N = 3). Esophageal cancer Kyse-30 cells resulted in 35.1% ± 1.6, (N = 3) of capture efficiency. Lastly, within pancreatic cancer cell lines, the capture efficiency of BxPC-3 was higher (33.80% ± 4.8, N = 3) than PANC-1 (26.80% ± 3.6, N = 3).

### 3.2. Immunocytochemistry Studies

The experimental conditions to be adopted in the analysis of patient samples in the RUBYchip™ were optimized through several immunocytochemistry experiments. CTC detection immunoassay conditions for the RUBYchip™ were optimized using both cultured adherent cells and cells in suspension trapped inside the microfluidic device.

**Figure 7:**
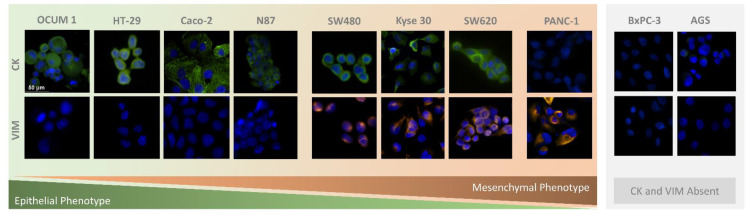
Microscopic images obtained from immunocytochemistry assays in adherent colorectal (SW480, SW620, Caco-2, HT-29), pancreatic (PANC-1, BxPC-3), gastric (N87, OCUM 1, AGS), and esophageal (Kyse 30) cells.

**Figure 8:**
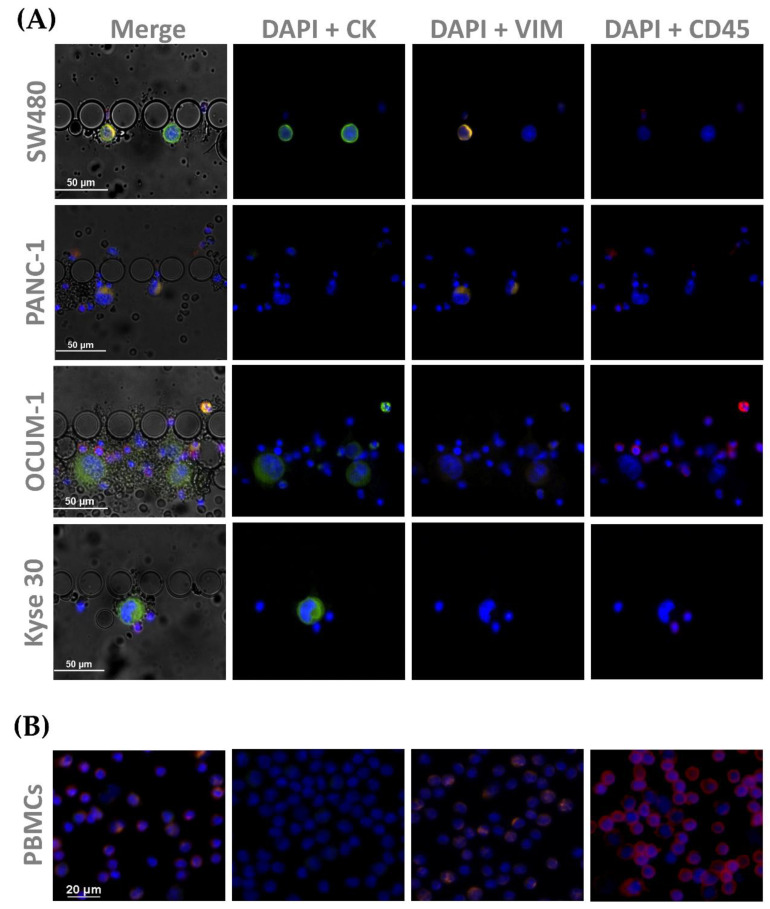
Microscopic images obtained from immunocytochemistry assays in (**A**) spiked cells trapped inside the device, with cell lines from each tumor type (SW480, PANC-1, OCUM 1 and Kyse 30); and (**B**) isolated PBMCs as a control All cells were stained for CK (1:200), and VIM (1:50), as well as CD45 (1:50).

CTC phenotyping was carried out using common EMT biomarkers, CK and VIM. Their respective expression patterns in all cancer cell lines are presented in Figure 7 and Figure 8A. Overall, CRC, esophageal and gastric cell lines showed CK expression, except for gastric AGS cells. Concomitant with CK expression, SW480, SW620 and Kyse 30 cells also displayed Vimentin expression. These results are in accordance with previous reports [35,36,37]. Regarding pancreatic cell lines, CK expression was absent from both BxPC-3 and PANC-1 cells, however VIM expression was present in PANC-1 cells. In accordance, others have already reported that several EMT markers were identified as being differentially expressed in BxPC3 and PANC-1 cells, indicating that PANC-1 cells show decreased expression of epithelial markers and increased expression of mesenchymal markers, which closely resembles the EMT phenotype [38]. Additionally, control PBMCs labelled with the same cocktail of antibodies showed mild VIM expression, as well as clear CD45 expression, as expected (Figure 8B).

Optimized antibody dilutions were established to be applied in the analysis of further patient samples.

### 3.3. CTC Enumeration in Gastrointestinal Cancer Patients’ Samples Using the RUBYchip™

A total of 11 GI cancer patients (3 CRC, 3 GC, 3 EC and 2 PC) followed at IPO-Porto, were recruited to participate in this study. All patients had advanced disease presenting locoregional and/or distant metastasis, as presented in Table 2.

Whole blood samples of 7.5 mL were collected from each patient prior to treatment initiation at IPO-Porto, to be subsequently used for CTC analysis, as described in the Section 2. Briefly, CTCs were defined as nucleated cells (determined by DAPI staining) expressing either CK or Vimentin and lacking the marker of hematopoietic lineage CD45 (CK+/CD45− and/or VIM+/CD45−). Cells that were CD45-positive (CK−/VIM−/CD45+) were classified as WBCs. Occasionally, double stained cells for CK and CD45 (CK+/CD45+) were observed. These were also classified as WBCs based on their multi-lobed nucleus and morphologies similar to neutrophils. Using this classification, phenotypically different CTCs were detected in all tested patients and CTC counts ranges from 1 to 44 CTCs/7.5 mL (an average of 9.72 CTCs/7.5 mL sample) (Table 3).

The CTCs isolated in this patient cohort displayed varying CK and VIM expression. In fact, 64% of the total isolated CTCs did not express CK at all, and most were revealed to have a mesenchymal phenotype (VIM+/CD45−). Only 26% of the captured CTCs were CK positive (CK+/CD45−) and 10% showed simultaneous CK and VIM expression (CK+/VIM+/CD45−), representing EMT CTCs. Across most GI cancer types, there were several patients presenting a predominantly mesenchymal CTC phenotype. Over ≥75% mesenchymal CTCs enumerated, this was observed in CRC (P2), GC (P4, P5, P6) and EC (P8 and P9). Despite not including PC, in fact the mesenchymal phenotype is also significant in this cancer type, as mesenchymal CTCs comprised 47% to 50% of total CTCs (P10 and P11).

**Figure 9:**
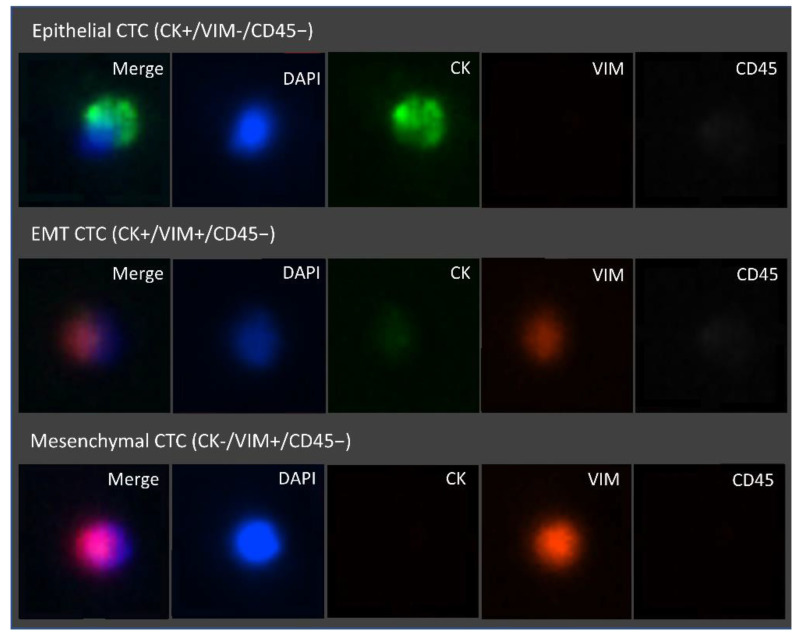
Representative fluorescence microscopic images of the different CTC phenotypes trapped inside the microfluidic device. Isolated cells trapped were stained with FITC Anti-Cytokeratin, Alexa Fluor 647 Anti-CD45, eFluor 570 Anti-Vimentin, and the nuclear stain DAPI. From top to bottom, epithelial (DAPI+/CK+/VIM−/CD45−), epithelial to mesenchymal transition (DAPI+/CK+/VIM+/CD45−) and mesenchymal (DAPI+/CK−/VIM+/CD45−) CTCs are represented. Images were acquired and observed with a 10×objective.

Interestingly, P4 (GC) was the highest CTC count observed, and, besides having a prevalent mesenchymal phenotype, it also presented the highest percentage of EMT CTCs of all cases (20.5%). Together, these results show that the studied GI cancer types present distinctive distributions of the CTC subpopulations in relation to the EMT phenotypic spectrum. Furthermore, the RUBYchip™ was equally efficient capturing the different CTCs phenotypes, as shown in Figure 9. 

## 4. Discussion

In this study, we describe the use of a microfluidic device for the label-free isolation of CTCs from patients with GI cancer. We found that the RUBYchip™ can efficiently isolate GI cancer CTCs based on their size, while depleting WBCs based on their deformability. The device design and geometry were ideal to allow the larger and less deformable cells to be retained in the filter gaps. First, the highest capture efficiencies (60–70%) were observed in cancer cells with larger dimensions (Caco-2 and SW480), whereas smaller cells were less retained. Still, apart from the physical features, there are intrinsic biological properties of the cancer cell models (mechanical stress tolerance, cytoskeleton stiffness, cell cycle stage, etc.), that may impact capture efficiency outcomes. Moreover, it is described that CTCs may have a wide range of cell sizes and diverse phenotypes. Thus, we have included ten different GI human carcinoma cell lines which differed in tumor model, cell size and phenotype. This allowed us to experimentally mimic a wide and reliable representation of what GI cancer CTCs may be like in circulation in real clinical samples [39]. Secondly, a consistent cell capture ability of the device was demonstrated, since altering the target number of spiked cells does not affect the capture efficiency, which suggests that the capture efficiency of the device will be equally maintained, regardless of the number of CTCs contained in the sample. Thus, we have demonstrated that the RUBYchip™ is able to isolate a diversity of cells, including those undergoing epithelial-to-mesenchymal transition.

Including clinical samples in this pilot study provided a proof-of-concept demonstration that the RUBYchip™ is able to efficiently isolate GI CTCs with high efficiency. CTCs were detected in all clinical samples processed, and 73% of analyzed cases were ≥3 CTCs. While some CTC technologies require manual and time-consuming pre-processing steps [40,41], whole blood samples can be directly processed with this microfluidic technology, hence limiting the loss of the very rare CTCs.

Regarding CRC, CTC counts above or equal to 3 CTCs per 7.5 mL of blood were used as a cutoff value in several studies as a positive marker to determine high-CTC patients using the CellSearch^®^ system [42,43,44]. These studies reported a significant correlation between baseline high CTC status and reduced survival. For patients undergoing surgery, CTCs counts above or equal to 1 CTC per 7.5 mL of blood were used by Bork et al. and van Dalum to determine survival, suggesting a reduced surgical utility for patients above the prognostic threshold [45,46]. Similar findings were observed in esophageal and pancreatic cancer. Overall, CTC-positivity was significantly associated with poor progression-free survival and overall survival (OS). Thus, the risk of death and tumor progression was higher in CTC-positive patients than in CTC-negative patients [47,48]. Currently there is no consensus on the optimal cutoff of CTCs for predicting the clinical outcome of pancreatic cancer. However, several studies using the standard technology used a cutoff of ≥1 CTC/7.5 mL to show poorer clinical outcomes than those without detectable CTCs [49,50].

Other studies have reported that the detection of CTCs in the peripheral blood of GI cancer patients may have clinical utility in monitoring tumor recurrence and metastatic spread [51,52]. According to a meta-analysis study encompassing 26 trials, the detection of CTCs was significantly related with the patients’ OS in all stages of GC [53]. Another prospective trial with gastric patients observed that patients with CTCs ≥ 5/7.5 mL detected in postoperative blood samples, using the CellSearch^®^ system, had significantly lower disease-free survival and OS than those with smaller numbers of CTCs [54]. Currently, in GC, there is no optimal cutoff value for CTCs in the peripheral blood for predicting prognosis. Still, most studies used the cutoff value of CTCs ≥ 1/7.5 or ≥ 2/7.5 mL and they reported that CTC positivity at both cutoff values was strongly correlated with worse prognosis. Other studies, using the CellSearch^®^ system, conducted by Li et al. and Matsusaka et al. showed that the cutoff value of CTCs ≥ 3/7.5 or ≥ 4/7.5 mL was also associated with poor prognosis in these patients [42,55].

Even though the metastatic process is not yet fully understood, it is widely known that EMT could contribute to the generation of CTCs by facilitating intravasation into the blood circulation. EMT is a process characterized by the upregulation of mesenchymal markers such as vimentin and, simultaneously, the downregulation of epithelial markers like cytokeratin or EpCAM. It has been widely correlated with disease aggressiveness, resistance to therapy and decreased PFS and OS, and it may underlie the biology of tumor dissemination and treatment resistance [56]. In fact, mesenchymal or EMT CTCs have already been reported to be associated with disease progression and with the presence of distant metastasis in different malignancies [57,58]. Hence, the identification of CTCs according to their EMT phenotypes may provide valuable information in the clinical setting.

Moreover, immunostaining for markers such as CK and VIM enabled the observation of different CTC subpopulations in this clinical cohort, including epithelial (CK+/VIM−/CD45−), EMT (CK+/VIM+/CD45−) and mesenchymal (CK−/VIM+/CD45−) CTCs. Remarkably, more than half (64%) of the cases in this clinical cohort displayed a prominent mesenchymal phenotype (≥50% mesenchymal CTCs). This highlights the relevance of using a label-free approach for CTCs isolation, which warrants an effective isolation of mesenchymal and EMT CTCs.

Similar to what we observed in clinical samples, another study, using the CanPatrol™ system, has also shown the presence of CTCs bearing a mesenchymal phenotype, which highlights the heterogeneity present in the CTCs from GC patients. Interestingly, they also observed an obvious increase in the number of mesenchymal CTCs in late-stage gastric cancer [59]. While very limited clinical correlations can be drawn from a small clinical cohort, there is growing evidence that the expression of EMT markers in CTCs may indicate later stage, more aggressive disease and correlate with a worse prognosis in different malignancies, such as hepatocellular and pancreatic tumors. However, these observations needs further clinical evaluation [60,61,62].

It is worth mentioning that the only FDA-cleared technology for CTCs capture and analysis in the clinic is widely dependent on epithelial marker expression, which has been limiting CTCs studies in EMT contexts. Thus, our label-free technology can overcome these limitations and help to elucidate the EMT process, as well as to clarify cancer metastasis mechanisms. This microfluidic platform not only achieves high performance in cell isolation, but it also enables a simple workflow and fast processing time while preserving cell integrity.

## 5. Conclusions

Mirroring previous findings from our group [31], it was demonstrated that the largest and least deformable cells were captured with high sensitivity using the RUBYchip™. We have also shown that CTCs can be efficiently isolated in less than 1 h and in a label-free manner from patients with metastatic GI using this technology. It is noteworthy, that in clinical samples, a considerable fraction of the isolated CTCs was negative for classical epithelial biomarkers and positive for EMT biomarkers. Furthermore, mesenchymal CTCs were found in 10 out of the 11 GI cancer patients. In fact, for most cases the mesenchymal phenotype was prevalent over the epithelial phenotype, particularly in gastric cancer patients.

First, these results show that this microfluidic chip can efficiently isolate cells that are undergoing epithelial-to-mesenchymal transitions. Secondly, the results uncover the power of the label-free isolation method described here, enabling further research intended at the validation of EMT CTCs as biomarkers for the non-invasive monitoring of cancer progression. Phenotyping CTCs subpopulations can potentially assist in the stratification of patient populations at risk and ultimately guide individual clinical and therapeutic decisions, enabling personalized medicine. Therefore, in the future, we aim to further validate our findings in a larger longitudinal cohort of metastatic GI cancer patients to evaluate the prognostic value and disease monitoring ability of the RUBYchip™.

## 6. Patents

The RUBYchip™ design is based on the patent PCT/EP2016/078406, filed by INL in front of the EPO on 22 November 2016, covering the geometry of the microfluidic system for CTC isolation, and currently licensed exclusively to RUBYnanomed.

## Figures and Tables

**Table 1:** Cell dimensions of each gastrointestinal cancer cell lines (cell size range, average cell size and average NC ratio, N = 10).

Cell Line	Cell Size Range (µm)	Average Cell Size (µm)	Average NC Ratio
Caco 2	14.94–35.17	22.80	0.68
OCUM 1	14.38–30.31	22.20	0.67
Kyse 30	15.99–20.96	20.62	0.67
PANC-1	12.82–23.82	19.91	0.68
BxPC-3	11.65–25.73	19.17	0.69
SW480	13.01–24.39	18.01	0.77
SW620	12.00–22.23	15.59	0.74
AGS	13.25–17.74	15.25	0.72
N87	9.84–19.30	13.23	0.78
HT-29	8.75–21.10	13.03	0.80

**Table 2:** Clinicopathological characteristics of patients enrolled in this study.

Patient Clinical Data	N
**Cohort**	
Number of patients	11
**Tumor type**	
Colorectal Cancer	3
Gastric Cancer	3
Esophageal Cancer	3
Pancreatic Cancer	2
**Sex**	
Female	2
Male	9
**Average age at sample collection**	58
**Disease Stage**	
**Tumor (T)**	
T1	1
T2	0
T3	4
T4	4
Tx	2
**Lymph node metastasis**	
N0	0
N1	2
N+	8
Nx	1
**Distant metastasis (M)**	
M0	5
M1	4
Mx	2
**CTC positive patients**	11

The labels T 1,2,3,4 refer to the size and extent of the primary tumor. The higher the number after the T, the larger the tumor or the more it has grown into nearby tissues. Tx: Main tumor cannot be measured. N0: There is no cancer spread in nearby lymph nodes. N1: Number of nearby lymph nodes that cancer has spread. Nx: Cancer spread in nearby lymph nodes cannot be measured. N+: Clinical evidence of lymph nodes metastasis. M0: Cancer has not spread to other parts of the body. M1: Cancer has spread to other parts of the body; Mx: Metastasis cannot be measured.

**Table 3:** CTC enumeration per 7.5 mL of whole blood samples from GI cancer patients.

Patients	Tumor Type	Disease Stage	CTC Enumeration (Baseline)				
			Epithelial	EMT	Mesenchymal	Total	
P1	CRC	TxNxM1	2	0	0	2	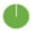
P2	CRC	T4N1M1	1	0	4	5	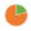
P3	CRC	T3a/bN+Mx	2	0	1	3	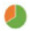
P4	GC	T4aN+M1	2	9	33	44	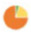
P5	GC	T3/4aN+M0	1	0	5	6	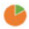
P6	GC	T3/4N+M0	0	0	5	5	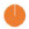
P7	EC	T1N1M0	6	0	3	9	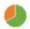
P8	EC	T3N+Mx	0	0	1	1	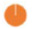
P9	EC	T4bN+M0	0	0	2	2	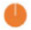
P10	PC	TxN+M0	8	0	7	15	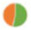
P11	PC	T4N+M1	5	1	6	12	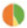

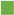
 Epithelial CTC; 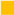 EMT CTC; 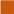 Mesechymal CTC.

## Data Availability

Not applicable.
